# Simultaneous impact of atorvastatin and mesenchymal stem cells for glioblastoma multiform suppression in rat glioblastoma multiform model

**DOI:** 10.1007/s11033-020-05855-z

**Published:** 2020-09-27

**Authors:** Arash Goodarzi, Mehdi Khanmohammadi, Arman Ai, Hamid Khodayari, Armin Ai, Morteza Sagharjoghi Farahani, Saeed Khodayari, Somayeh Ebrahimi-Barough, Sanam Mohandesnezhad, Jafar Ai

**Affiliations:** 1 Department of Tissue Engineering and Applied Cell Sciences, School of Advanced Technologies in Medicine, Fasa University of Medical Sciences, Fars, Iran; 2 Skull Base Research Center, The Five Senses Institute, Hazrat Rasoul Akram Hospital, Iran University of Medical Sciences (IUMS), Tehran, Iran; 3 School of Medicine, Tehran University of Medical Sciences, Tehran, Iran; 4 International Center for Personalized Medicine, Dusseldorf, Germany; 5 Breast Disease Research Center (BDRC), Tehran University of Medical Sciences, Tehran, Iran; 6 Scientific Research Center, School of Dentistry, Tehran University of Medical Sciences, Tehran, Iran; 7 Cancer Research Center, Tehran University of Medical Sciences, Tehran, Iran; 8 Department of Tissue Engineering and Applied Cell Sciences, School of Advanced Technologies in Medicine, Tehran University of Medical Sciences, Tehran, Iran

**Keywords:** Glioblastoma model, Atorvastatin, Mesenchymal stem cells, Tumor suppression

## Abstract

Glioblastoma multiform (GBM) is known as an aggressive glial neoplasm. Recently incorporation of mesenchymal stem cells with anti-tumor drugs have been used due to lack of immunological responses and their easy accessibility. In this study, we have investigated the anti-proliferative and apoptotic activity of atorvastatin (Ator) in combination of mesenchymal stem cells (MSCs) on GBM cells in vitro and in vivo. The MSCs isolated from rats and characterized for their multi-potency features. The anti-proliferative and migration inhibition of Ator and MSCs were evaluated by MTT and scratch migration assays. The annexin/PI percentage and cell cycle arrest of treated C6 cells were evaluated until 72 h incubation. The animal model was established via injection of C6 cells in the brain of rats and subsequent injection of Ator each 3 days and single injection of MSCs until 12 days. The growth rate, migrational phenotype and cell cycle progression of C6 cells decreased and inhibited by the interplay of different factors in the presence of Ator and MSCs. The effect of Ator and MSCs on animal models displayed a significant reduction in tumor size and weight. Furthermore, histopathology evaluation proved low hypercellularity and mitosis index as well as mild invasive tumor cells for perivascular cuffing without pseudopalisading necrosis and small delicate vessels in Ator + MSCs condition. In summary, Ator and MSCs delivery to GBM model provides an effective strategy for targeted therapy of brain tumor.

## Introduction

Within a comparison view to the last global cancer statistics, an increasing trend into the prevalence of central nervous system (CNS) tumors and also its related death has been observed [1–4]. In this regard, the CNS tumors respectively show 1.6 and 2.5 percentages of all new cancer cases and mortalities just in 2018 [4]. Among these, glioblastoma multiform (GBM) as one of the most extensive and common type of the CNS malignancies holds poor therapeutic responses and also short median survival [1, 5–7]. Although multimodal aggressive therapies have been developed in order to the treatment of GBM cases. It should be noted that GBM patient’s median survival is not much more than 18 months after the diagnosis. Local radiotherapy and also systemic chemotherapy in combination of surgical resection are the main conventional GBM therapeutic approaches [4, 7–9]. However, due to the high-raise of mortality in GBM patients and the low efficiency of conventional medications, developing of the new and safe therapeutic strategies with a more anti-tumoral efficacy are required [4, 6–9]. It has clearly understood that the GBM progression and correspondingly mutual patient’s therapeutic responses are significantly affected by the GBM specific microenvironment [10–14]. Generally, the GBM microenvironments appear through secretion of several pro-inflammatory cytokines as well as hypoxic condition [15–18]. In this process, as the main pro-inflammatory microenvironment mediators including interleukin-1β (IL-1β), interleukin-6 (IL-6), and also interleukin-8 (IL-8) increase by activation of their related downstream targets such as the JAK-STAT, PI3K-AKT, and mitogen-activated protein kinase (MAPK) signaling pathways [7, 9, 19–21]. These conditions cause GBM cell proliferation, invasion, migration and also tumor angiogenesis within the brain region [3, 5–7, 19, 21]. Herein, it has been suggested that the GBM specific microenvironment components such as anti-GBM drugs and also their related inhibiting downstream pathways activated by stem cells are able to be as a high potent targets in order to develop the novel GBM therapeutic approaches [4, 6, 8, 22–24].

Nowadays, mesenchymal stem cells (MSCs) have been introduced as one of the effective mammalian’s cellular populations to manage the malignant tissues behaviors [6, 8, 22, 23]. As a type of multipotent cells, beside their self-renewal and differentiation potential, it has been demonstrated that the MSCs have ability to express an array of paracrine/autocrine factors through secretion of anti-inflammatory molecules [6, 22, 23]. Previous observations described that the direct interaction of MSCs within some tumor’s complicate microenvironment, such as the GBM, can actively regulate the tumor’s immunological responses and in following inhibit the malignancy progression [6, 10, 12, 22, 23, 25]. Besides the modulation of the GBM inflammatory microenvironment, it resembles that the interactions of the MSCs through activation of tumor necrosis factor-related apoptosis-inducing ligand (TRAIL), as one of the central cell death ligands, progress apoptosis process into cancerous cells [6, 10, 12, 22, 23, 25].

On the other side, during the past decades, statin family, as kinds of HMG-CoA inhibitors have been found an extensive medical application in treatment of diverse types of disorders and diseases [3, 9, 11, 16, 20, 21]. For instance, atorvastatin (Ator) as a kind of the statins, with its impactful role in curing some types of cardiovascular disease has known as an appropriate therapy approach in inhibition of photogenic vessels and angiogenesis development [3, 9, 11, 16, 20, 21]. It seems that Ator is stimulus anti-angiogenic response, notably into the inflammatory microenvironment which may be formed by down-regulation of some core antigenic factors including the vascular endothelial growth factor (VEGF), tumor necrosis factor-α (TNF-α), and also transforming growth factor-β1 (TGF-β1) [3, 9, 16, 21, 26]. Subsequently, it is expected that utilizing variety of statins may be a useful medication for hindering the malignant tissues progression [3, 9, 16, 21]. Accordingly, the main goal of our study was evaluation of beneficial effects of Ator in high-dose as well as MSCs on anti-GBM activities in experimental rat GBM models.

In the present study we aimed to evaluate the anti-proliferative apoptotic and cell cycle arrest effects of Ator and MSCS on C6 glioma cell line in vitro and their anti-tumor impact in vivo. The cellular proliferation, scratch migration, annexin/PI cell cycle and cell cycle arrest were evaluated for treated C6 cells with Ator and MSCs. The GBM model was established and Ator and MSCs were implanted in rat model until 12 days. The hematoxin and eosin staining of brain tissue and tumor size was evaluated to reveal anti-tumor activity of these substrates.

## Materials and methods

### Materials

Minimum essential medium eagle-alpha modification (α-MEM), dulbecco’s modified eagle medium (DMEM), fetal bovine serum (FBS) and penicillin-streptomycin were purchased from Gibco Life Technologies (Grand Island, NE, USA). Atorvastatin ([R-(R*, R*)-2-(4-Fluorophenyl)-ß, d-dihydroxy-5-(1-methylethyl)-3-Phenyl-4-(Phenylamino)-Carbonyl]-1-H-Pyr- role-1-heptanoic acid), dimethyl sulfoxide (DMSO), 3-(4,5-dimethyl-2-thiazolyl)-2,5-diphenyl-2H-tetrazolium bromide (MTT), trizol solution and 2 × Real Rime SYBER GREEN master mix, phosphate-buffered saline (PBS), ketamine and xylazine were purchased from Sigma-Aldrich (St. Louis, MO). Terminal deoxy- nucleotidyl transferase mediated dUTP nick-end labeling (TUNEL) Kit was obtained from (Roche, Mannheim, Germany).

### Study design

**Fig. 1:**
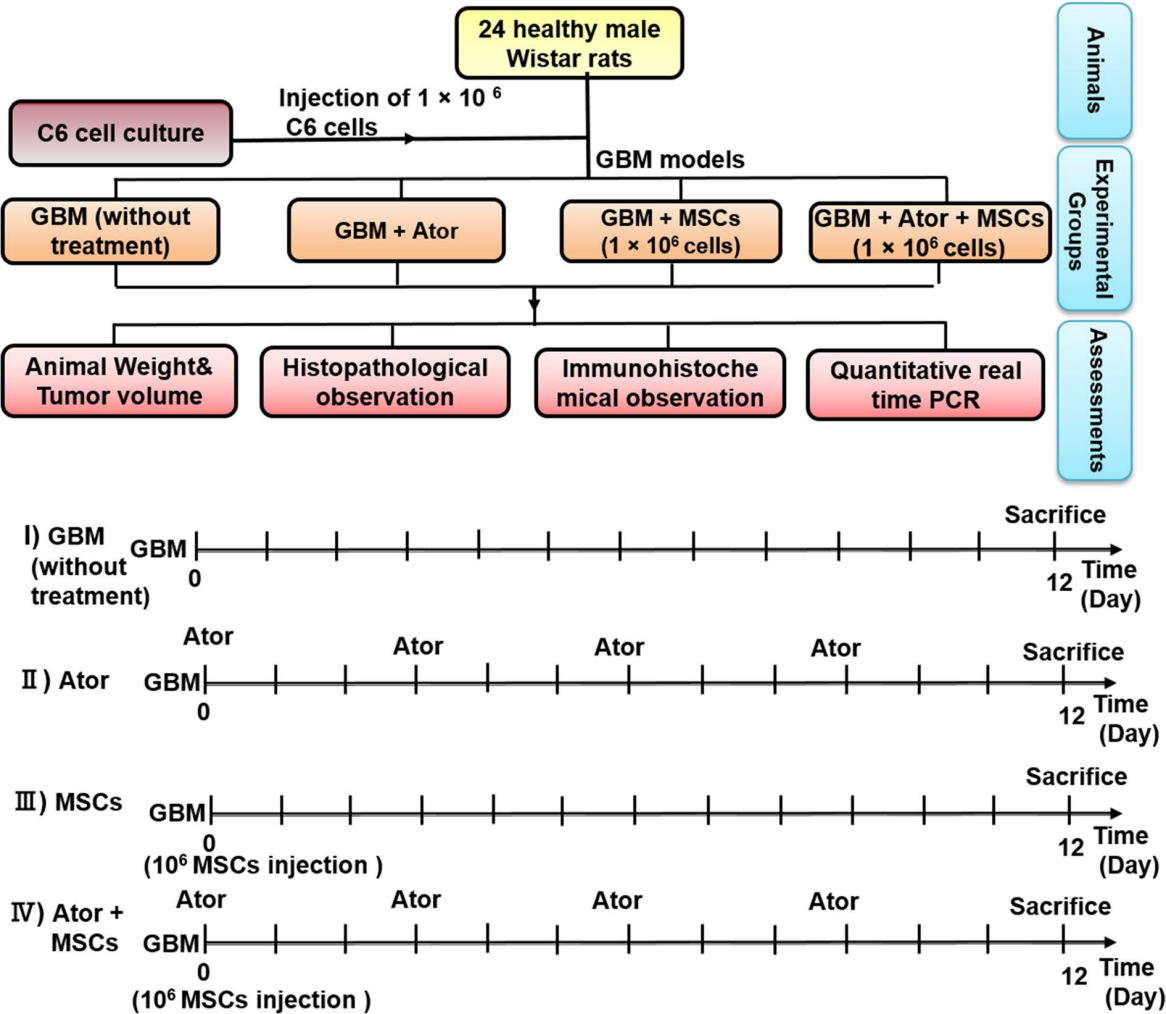
Schematic of study design for evaluation of atorvastatin (Ator) and mesenchymal stem cells (MSCs) impact in rat glioblastoma multiform (GBM) model

This study was conducted on four series of experiments including: first group, GBM model without any treatment, second group, the effect of Ator on glioblastoma cancer cell lines; third group, anti-tumorigenic and anti-metastatic effects of MSCs and fourth group simultaneous incorporation of Ator and MSCs on histopathology of animal model (Fig. 1).

### Animals

The male Wistar rats (200–220 g) of 2 months of age were purchased from Pasteur Institute (Tehran, Iran). Those preserved in ventilated and room temperature controlled at 23–25 °C with a 12 h light/dark cycle and had access standard food pellets and water. Animals use and care were approved with national ethics committee of Tehran University of Medical Sciences (ethical code: IR.TUMS.VCR.REC.13,951,833) and were performed in accordance with the university’s guidelines. Furthermore, all animal experiments comply with the National Institutes of Health guide for the care and use of laboratory animals (NIH Publications No. 8023, revised 1978).

### Cell culture

Bone marrow mesenchymal stem cells (MSCs) were isolated from wistar rats according previously described protocol [27]. Briefly, the wistar rats aged 6–8 weeks were terminated by cervical dislocation and soaked in 70% (v/v) ethanol and then the animal was transferred to a new dish. Tibias, femurs, and humeri were dissected by cutting at the joints, and the bones were transferred to a 100-mm sterile culture dish with 10 mL complete α-MEM medium on ice. Then two ends of femurs and tibias were cut open with a scissor and then 5 ml DMEM in syringe, flush the marrow into a 50 mL tube by inserting the 22-G needle to one open end of the bone. After that, marrows were obtained, re-suspended cells and were passed it through a 40 µm cell strainer to discard the blood aggregates as well as the bone debris. The cells were harvested via centrifugation process and were sub-cultured in 100 mm culture dish in a 37 °C and 5% CO_2_ incubator for 2 weeks. Medium was changed every 2 ~ 3 days. All samples were processed within 30 minutes following animal death to ensure high cell viability. The C6 cell line were purchased from the Cell Bank of the Pasteur Institute of Iran (Tehran, Iran). These cells maintained in DMEM medium containing 10% FBS and 1% penicillin-streptomycin.

### MSC phenotype

The expression of cell surface protein was investigated by flow cytometry technique. The MSCs were trypsinized and incubated with specific PE- or fluorescein isothiocyanate-conjugated (FITC)-conjugated anti-CD31, CD45, CD90, and CD105 fluorescent dye conjugated anti-human antibodies for 30 min at 4 °C in a dark place. Afterwards, the cells were washed and re-suspended in PBS. The suspended cells were applied in FACS caliber (BD Biosciences) for cell surface protein expression measurement. Flowcytometric data was analyzed using FlowJo software (FlowJo, LLC). Positive and negative controls were also evaluated in each run. Furthermore, to confirm the mesenchymal phenotype of isolated cells, these cells were differentiated into osteogenic and adipogenic lineages as previously described [28]. The cells were sub-cultured and passage-3 MSCs were used for all experiments.

### MTT assay

MTT assay was performed to measure anti-proliferation impacts of Ator and MSCs by MTT Kit on GBM tumor cell model [28]. Concisely, C6 cell lines at 2 × 10^4^ cells/48 well-plates were planted in for 12 h. Then medium was changed to fresh DMEM medium supplemented with Ator 100 µM and MSCs in equal density of C6 cells using 48-transwell plates with a polycarbonate filter (8-µm pore size, Millipore) until additional 72 h. At the indicated time points, 200 µL MTT solution 5 mg/mL was added in each well and incubated in a 5% CO_2_ and 37 °C. After 4 h, the medium was removed and insoluble purple formazan product was dissolved by DMSO for 15 min. The absorbance was measured at 570 nm was determined by a microplate reader (EL340 Bio-Tek Instruments, Hopkinton, MA).

### Cell migration assay

In this method, the C6 cells were seeded at a density of 2 × 10^4^ cells/cm^2^ were incubated for 24 h in a 48 well-plates [29]. The scratch was created on cells with 90% confluent monolayer mechanically with a sterile plastic yellow tip to make a uniform cell free area. Detached cells and debris were washed away with PBS. After that, cells were treated and incubated with Ator 100 µM or equal density of MSCs using 48-transwell plates as well as combination of both of Ator 100 µM and MSCs MSCs using transwell plate. Cell motility and migration into the wound area were monitored for 48 h using an optical microscope (Nikon Eclipse TE2000-S, Germany). The microphotographs were correlated and the number of cells migrated into the scratch was counted for quantitative analysis using Image J analysis software.

### Cell cycle assays

The C6 cells at 1 × 10^5^ cells/well were seeded in 6-transwell plates. After 24 h, attached cells were treated with different conditions including Ator 100 µM or MSCs using 6-transwell plates in equal density of seeded C6 cells as well as a combination of both of Ator and MSCs using transwell plate. After another 48 h, the conditioned medium containing floating C6 cells was collected and combined with adherent cells that were detached by brief trypsinization. The harvested cells were rinsed with ice-cold PBS, re-suspended in 0.5 mL of 70% ethanol overnight. Then, the cells were pelleted via centrifugation at 2000 rpm/5 min, re-suspended in 500 µL of PBS containing 50 µg RNase A and 5 µg propidium iodide (PI), and incubated in dark at 25 °C for 0.5 h. The cells were fully re-suspended and cell cycle was analyzed using a BD Accuri C6 flow cytometer (BD Biosciences, CA, USA). The obtained records were processed using AXP acquisition and analysis software.

### Annexin-PI assay

Annexin-PI assay was used to detect the percentage of C6 cells in apoptosis phase which induced by Ator and MSCs likewise cell cycle study. After 48 h treatment of C6 cells with Ator and MSCs, the annexin in and PI reagent were added to harvested C6 cells and kept in the dark on ice for 30 min. Then C6 cells were washed via PBS and centrifugation to removed non-reacted fluorophore molecules. The fluorescence of labeled cells was measured using a flow cytometer.

### GBM model establishment

Twenty four wistar rats were obtained, weighed and divided into four groups to study in vivo phase (Fig. 1). To establishment of the GBM, the rats were anaesthetized with ketamine-xylazine mixture (120 mg/kg/IP) and positioned in a stereotaxic instrument (Stoelting, Hertfordshire, UK). The stereotaxic coordinates of the intracranial injection were AP = + 1.6 mm to bregma, ML = − 2 mm lateral to bregma, and DV = − 6 mm ventral. Animals were received C6 rat glioma cells at 5 × 10^4^ density in 2.5 µL DMEM through a single microinjection into the right stratum using 10 µL syringe (Hamilton, Bonaduz, Switzerland), at a constant rate of 0.5 µL/min. The experimental conditions were (I) GBM animal model (Control group), (II) GBM animal model treated with Ator group, (III) GBM animal model treated with MSCs group, and (IV) GBM animal model treated with combination of Ator and MSCs. In group IV 3 days after post-implantation of MSCs 1 × 10^6^ cells, rats were treated with Ator 100 µM three times with a 3-day interval. As shown in Fig. 1, animals were treated with MSCs 1 × 10^6^ cells by a single intracerebral injection, and an intraperitoneal injection of Ator 100 µM with an interval of 3 days between injections, according to the schematic illustration of the *in vivo* antitumor process.

### Antitumor efficacy assessment

The relative body weight and tumor size quantification were carried out during the treatment and end of study respectively. Tumor volume was calculated by summing the segmented regions. The malignant and normal tissue regions were completely distinguished by visual observations. The malignant tissues were dissected using a scalpel blade and weighed.

### Histological assay

After treatment for 12 days, the rats were euthanized and the tumors were collected and fixed in 4% PBS buffered paraformaldehyde followed by passage and embedding in paraffin. In order to perform the hematoxylin and eosin (H & E) staining, paraffin blocks were sectioned by 5 µm thickness. Slides were studied at the microscopic level (OLYMPUS-BX51 microscope), then an Olympus-DP12 camera was used to take digital photos.

### Quantitative real-time polymerase chain reaction

In order to define the expression level of VEGF, BCL2, BAX, IL1-β, BDNF, GFAP and KI67 genes, Trizol solution (Invitrogen, USA) was used to extract total RNA from tissues according to the manufacturer’s instructions. Afterward, cDNA was synthesized by RevertAid first-strand cDNA synthesis kit (Thermo Scientific Fermentas, USA), according to the manufacturer’s instructions. Primers for real-time PCR were designed using Beacon Designer 7 software. The primer sequences information presented in Table.1. Quantitative RT-PCR (qRT-PCR) was performed on cDNAs using Rotor-Gene Q 2plex HRM platform real-time PCR system (Corbett Life Science). The relative expression levels of anti- and pro- apoptotic gens were evaluated in comparison with GAPDH as an endogenous control gene. The RT-PCR reactions were done in duplicate, as performed in previous studies [3, 17]. Rotor-gene Q sequence detection system determined the threshold cycle (CT) values. Comparative threshold cycle (2 − ΔΔCT) method was used in order to analyze the data.

**Table 1:** List of primer couples generated for real-time RT-PCR

Gene	Primer sequence	
VEGF	F	AAGACCGATTAACCATGTCA
	R	ATGTCAGGCTTTCTGGATTA
BCL2	F	GTGGCCTTCTTTGAGTTCGG
	R	CACAGAGCGATGTTGTCCAC
BAX	F	CATCCACCAAGAAGCTGAGC
	R	GCAATCATCCTCTGCAGCTC
IL1-β	F	TTCAGGAAGGCAGTGTCACT
	R	CGTCTTGGTTTTGCAGCTCT
BDNF	F	AGCCTCCTCTGCTCTTTCTG
	R	CGCCGAACCCTCATAGACAT
GFAP	F	TAAGCGTCCATCCTCCTGTTTGAAAG
	R	ACACTAATCGAAGGCACTCCA
KI67	F	ATCAGACACAGTGACAGGCA
	R	TTGCATGCTCGTGACCTTCT

### Statistical analysis

All experiments were carried out in triplicate for each condition. The data are shown as means ± standard deviation (SD). Statistical analysis was done by Minitab 18 software (Minitab, Inc., State College, USA). Significant differences are expressed as *p < 0.05, **p < 0.01, ***p < 0.001 and ^#^p > 0.05 in bar graphs.

## Results

**Fig. 2:**
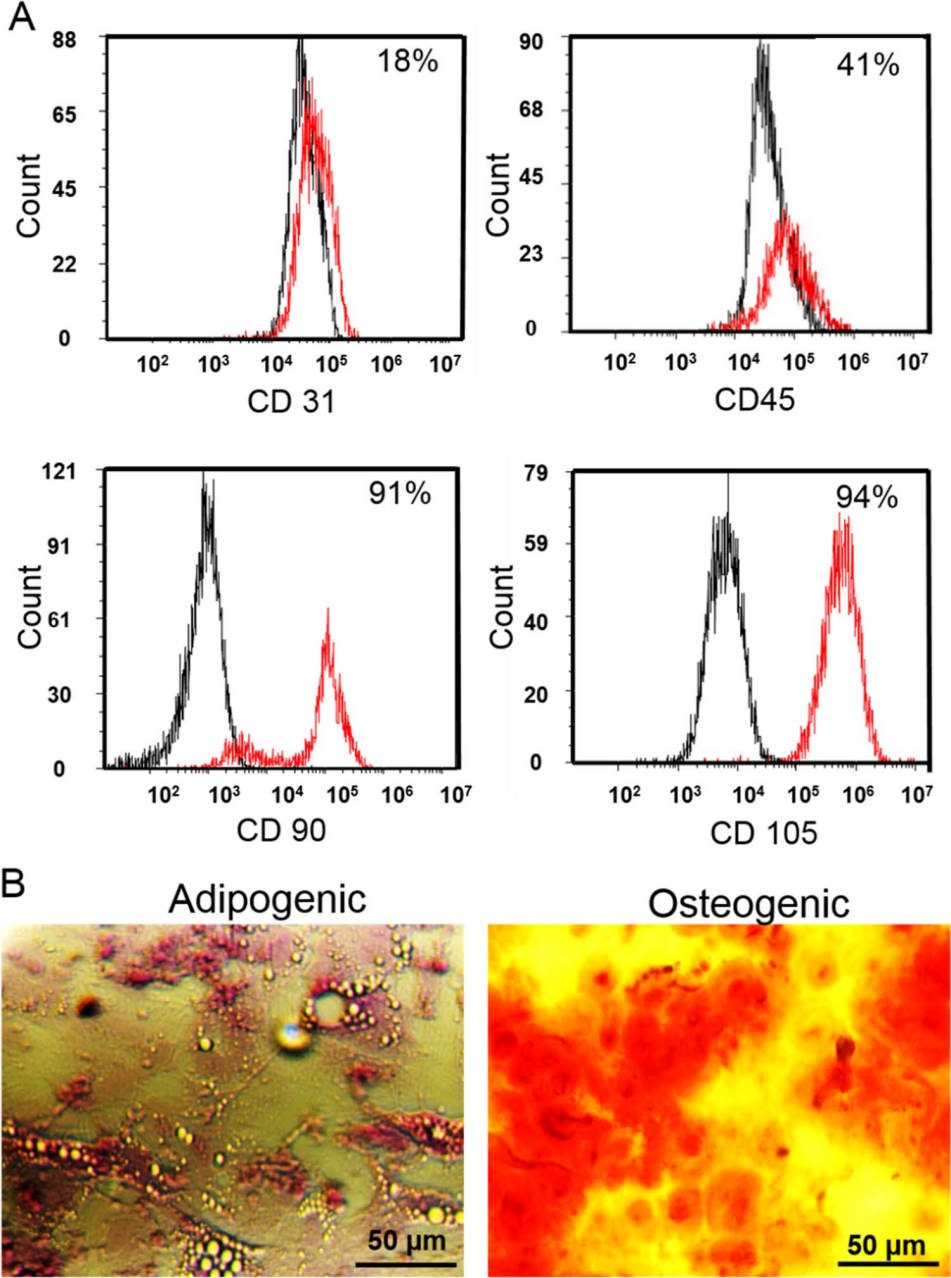
**a** Flow cytometry histograms of MSCs stained with fluorescein conjugated antibody. Black: MSCs without staining with corresponding fluorescein conjugated antibody; red: MSCs stained with corresponding fluorescein conjugated antibody. **b** Isolated MSCs differentiated into adipocytes and osteoblasts. (Color figure online)

The cells were mostly negative for CD31 at 11% and CD45 at 41% indicating nearly absence of the hematopoietic and endothelial cell markers among the isolated cells (Fig. 2a). Additionally, the cytofluorometric results revealed the expression of distinguished MSC CD markers including CD90 and CD105 antigens more than 90% (Fig. 2a). Moreover, the multi-lineage differentiation potential of isolated MSCs, as the major characteristic of mesodermal-originated cells, showed a number of lipid droplets as well as major calcium deposition after the adipogenic and osteogenic inductions respectively (Fig. 2b). Flow cytometry analysis of the MSCs with specific antibodies revealed the expression of prominent MSC markers including CD90 and CD105 and the low level of endothelial cell marker CD31 as well as hematopoietic stem cell marker CD45. Collectively, flow cytometry and differentiation potential of isolated cells verified the extracted bone MSC characteristics of the MSCs at the third and fourth passage [28, 30, 31].

### MTT assay

**Fig. 3:**
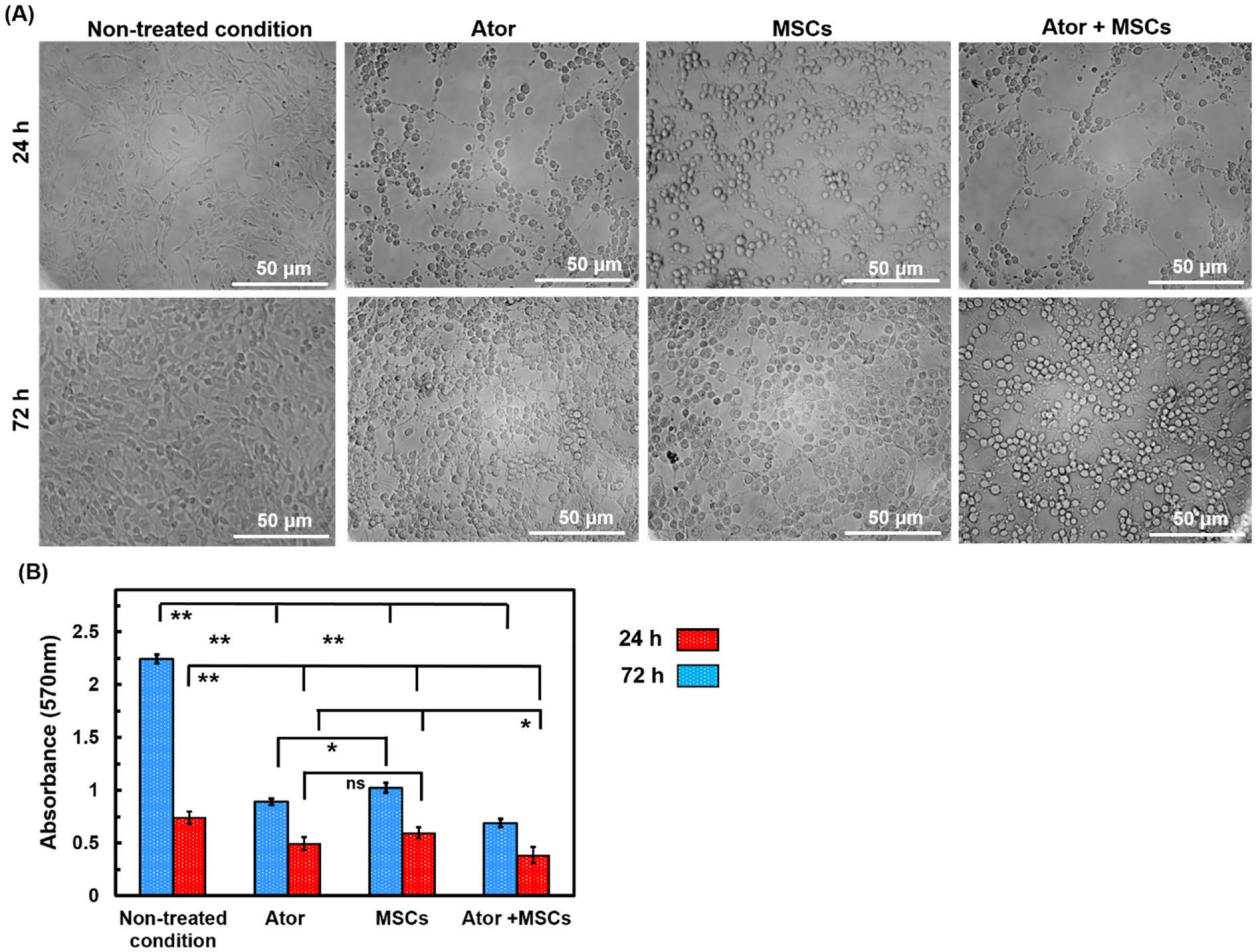
Cell viability of C6 Cells treated with Ator, MSCs and Ator + MSCs after 72 h. **a** Morphology observation of seeded C6 cells and **b** MTT assay histogram after 24 and 72 h treatment with Ator and MSCs. The results were expressed as mean ± SD

The growth potential of C6 cell after treating with Ator and MSCs was evaluated by cellular morphology observation and MTT assay. As can be seen, C6 cell viability increased in control group by increasing time of culture in spindle-shaped and cells were totally spread with cytoplasmic extension (Fig. 3a). Besides, cellular morphology was changed to spheroid-shape and growth suppression was observed by microscopic observation of cells during extended time of culture compared with non-treated condition. Meanwhile, high cell-free area was observed in treated condition with Ator and MSCs after 72 incubation time (Fig. 3a). The C6 cells were entirely covered plate surface in non-treated condition as a negative control group. An interesting point from Fig. 3 is that C6 cell viability significantly decreased in a time-depended manner with incorporation of Ator as well as MSCs (Fig. 3a and b). There were no substantial differences among cellular viability of Ator and MSCs groups at 24 h after incubation. The lower cell viability in Ator and Ator + MSCs conditions compared with alone MSCs condition was obtained at 72 h incubation (Fig. 3a and b). Recent studies show that Ator and MSCs affect mitochondrial functions of cells which has an important role in cell survival [17, 21, 23, 26, 32]. We found that Ator when combined with MSCs had more synergistic cytotoxicity on GBM cells about 60% lower cellular growth compared with single treated conditions with Ator or MSCs after 72 h.

### Migration assay

**Fig. 4:**
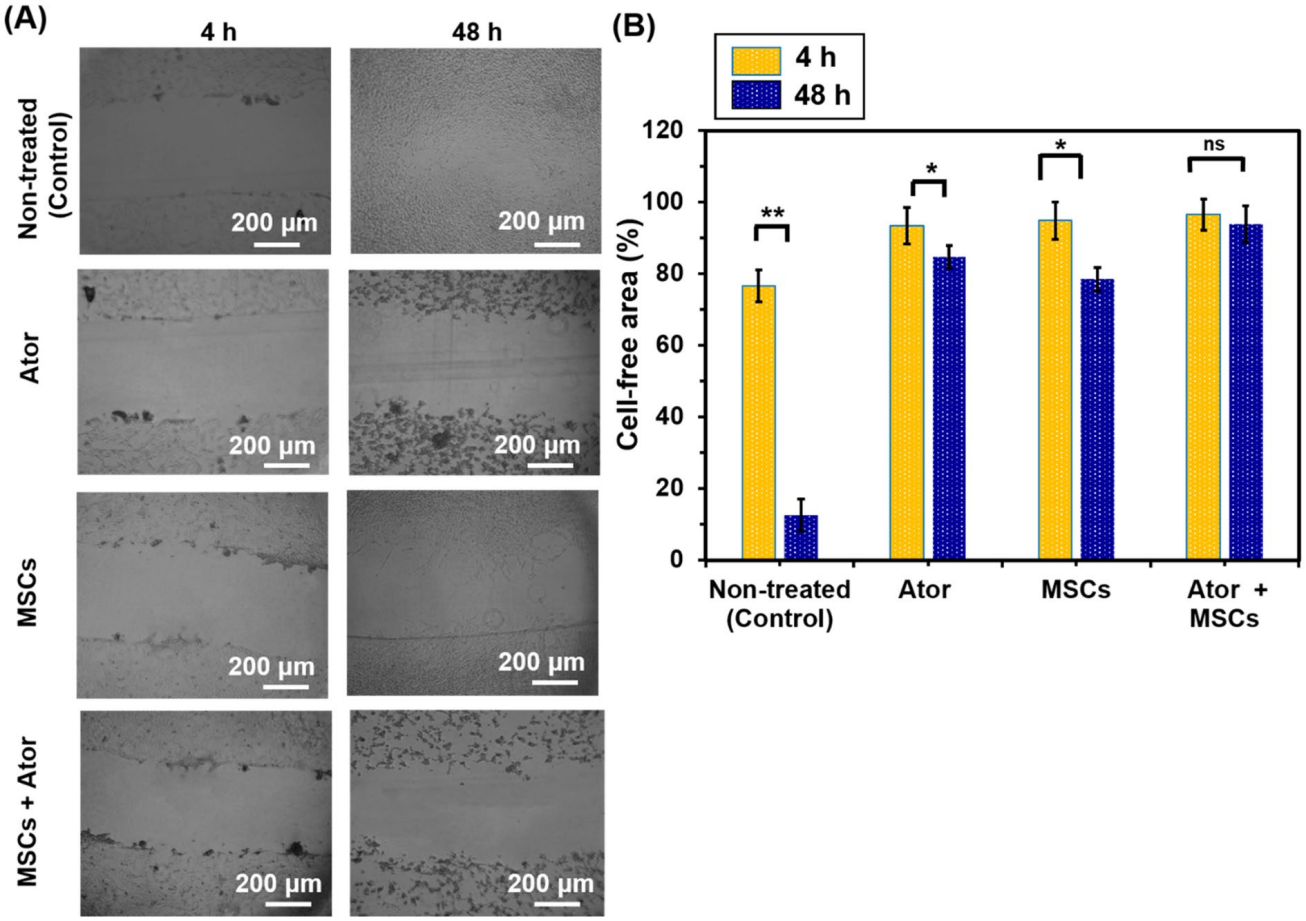
Migration of C6 cells after treatment with Ator, MSCs in transwellplate and combination usage of Ator and MSCs for 48 h

Scratch migration of C6 cells was evaluated according to the method described previously [29]. Figure 4 shows the morphology of C6 cells cultured on culture dish. We made successfully uniformly sized wounds of approximately 400 µm in width. The inhibitory effect of Ator, MSCs and Ator + MSCs on C6 cell migration was studied. The C6 cells covered the scratched area (cell-free area) of culture dish in control condition after 48 h of incubation (Fig. 4). Meanwhile, the cells covered only 54%, 73% and 43% of the scratched area of Ator, MSCs and Ator + MSCs conditions (Fig. 4a and b) (P < 0.05). The results indicated that Ator and Ator + MSCs had significant inhibitory effect on the invasion and migration of C6 cells (*p* < 0.05). In addition, cell migration level increased by increase of incubation time in experimental conditions proved that cell migration inhibition by Ator and MSCs was dose-time-dependent. Number of migrated and attached cells in the presence of MSCs was higher to some extent in comparison with Ator which proved effectiveness of Ator as revealed by cell proliferation study. Meanwhile, the cellular cytoplasm was condensed and cell size decreased from spindle shape to spheroid compared with control condition which cells were totally spread and cells had a long spindle-shaped morphology [3, 18, 19, 23]. There was no significant difference in C6 cell morphologies among these three conditions treated with tumor cell inhibitors during 48 h of culture. These data suggest that by Ator and MSCs treatment could obtain migration suppression and proliferation inhibition on C6 glioma cells. Whereas cultures of primary glial cells are observed in normal morphology and high migration rate when subculture in conventional culture system without exposure to anti-cancerous drug or stem cells.

### Annexin/PI and cell cycle

**Fig. 5:**
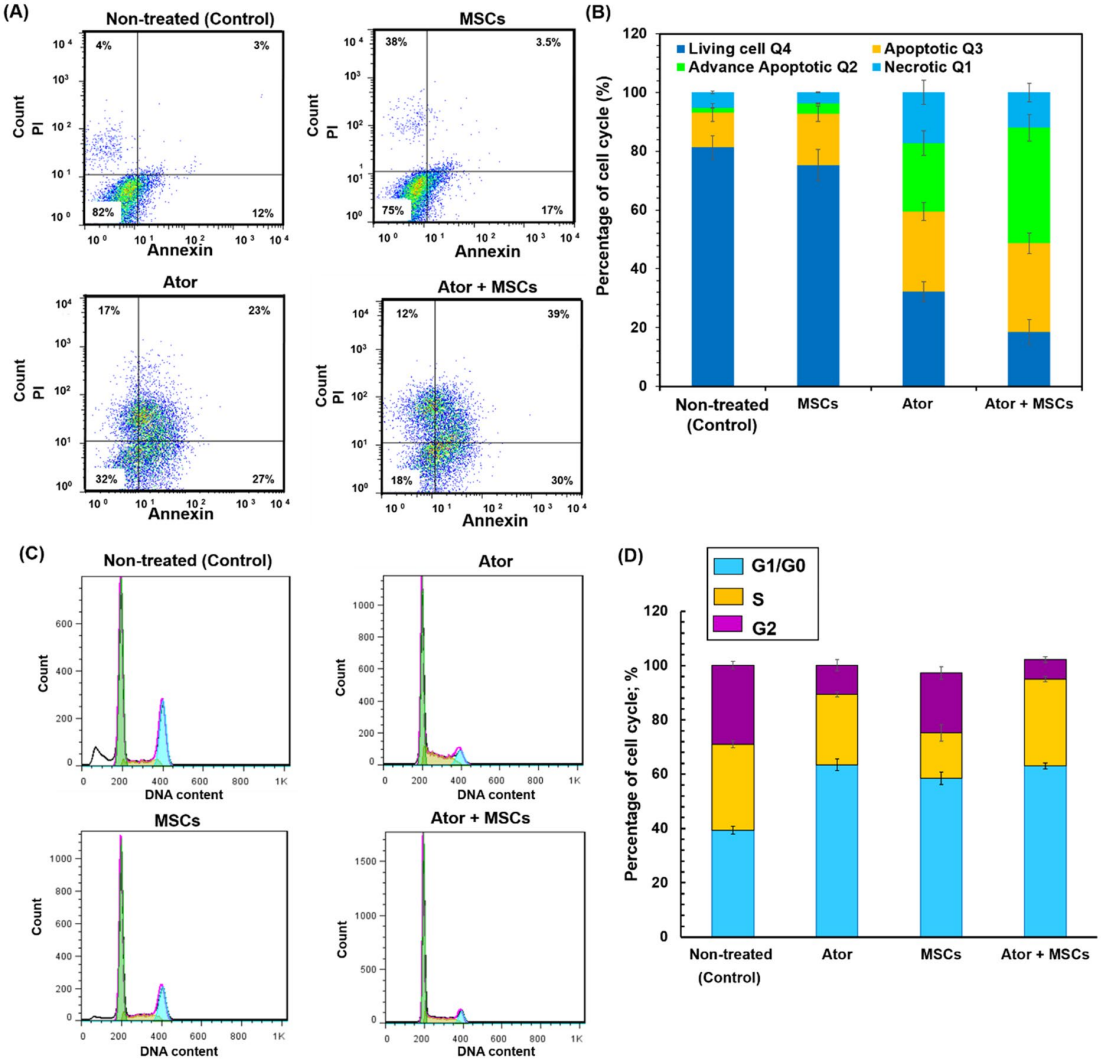
Flowcytometry assay to evaluate the effects of the MSCs, Ator and Ator + MSCs on the C6 cell apoptosis induction which compared with non-treated condition. **a** Histograms of harvested and stained C6 cells **b** Comparison of C6 cells death rates after 72 h treatment. Harvested C6 cells were stained with FITC-conjugated annexin V and propidium iodide and analyzed by the flowcytometry whereas annexin V and PI identify viable cells (annexin V−, PI−), early apoptotic cells (annexin V+, PI−), late apoptotic or necrotic cells (annexin V+, PI+) and necrotic cells (annexin V−, PI+). **c** Cell cycle distribution of C6 cells in different conditions including non-treated and treated by Ator, MSCs and combination of Ator with MSCs **d** Quantitative analysis of different cell phase populations. (Color figure online)

It is well known that the mitochondrial membrane loss mostly linked to the initiation and activation of apoptotic process in cells. The mitochondrial membrane damage and dissipation regulates intensification of apoptogenic factors and finally cell death [8, 9, 18, 19, 21, 23, 33]. In this study, apoptosis and necrosis degrees of C6 cells which induced by presence of Ator and MSCs were evaluated and compared non-treated C6 cells as a GBM model. The percentage of C6 cells in apoptosis and necrosis phases was assessed by flowcytometry for Ator, MSCs and Ator + MSCs experimental conditions (Fig. 5a and b). Early and late apoptosis rates of C6 cells were 4% and 3% respectively in non-treated condition (Control) (Fig. 5a and b). The early apoptosis and late apoptotic cells were respectively 17% and 3.5% for MSC and 27% and 23% for Ator condition (Fig. 5a and b). As can be seen in Fig. 5a and b the treated cells by Ator + MSCs had higher late apoptosis level and necrosis level compared to cells treated by Ator and MSCs as well as control group. For C6 cells treated with Ator + MSCs, majority of cells were in early and late apoptosis condition at 30% and 39% respectively (Fig. 5a and b). These results show that in the presence of Ator and Ator + MSCs were able to induce apoptosis faster in C6 cells as seen by the large amount of late apoptotic cells and diminished cell viability (Fig. 5a and b).

### Evaluation of cell cycle arrest

To investigate whether Ator and MSCs suppressed cancer cell proliferation through cell cycle arrest, we treated the C6 cells with Ator, MSCs and analyzed the cell cycle distribution by flow cytometry. Figure 5c and d show the histograms and percentage of cells in each phase of the cell cycle which were categorized into G1/G0, S, and G2/M phases. As can be seen form Fig. 5c and d utilization of Ator, MSCs and Ator + MSCs lead to stimulating G0/G1 cell cycle arrest and significant accumulation of C6 cells in G1/G0 phase of the cell cycle. The highest rate of G1/G0 arrest was found for Ator and Ator + MSCs conditions lower than 10%. Whereas, Ator and Ator + MSCs decreased cell population in the G_2_/M and S phases of cell cycle were about ∼2–4 times lesser than that of alone MSCs and non-treated conditions (Fig. 5c and d). These results confirmed that the Ator and MSCs incorporation properly persuaded C6 cell arrest in the G0/G1 phase of the cell cycle [6, 8, 9, 16, 19, 21, 23].

### Real-time polymerase chain reaction analysis

**Fig. 6:**
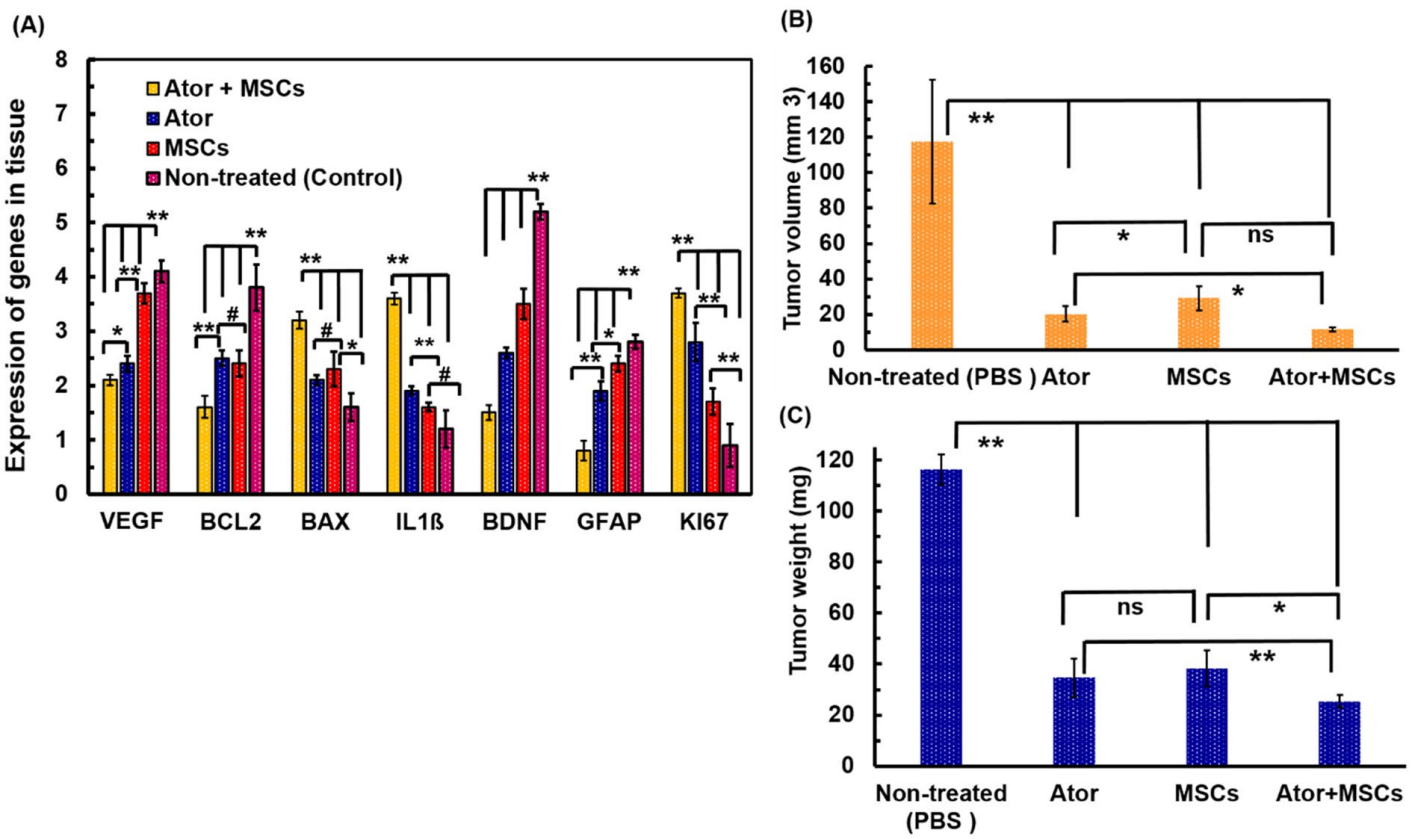
**a** Quantitative real time-PCR analyses of the mRNA Levels of angiogenesis markers (VEGF and BDGF), anti-apoptotic genes (BCL2 and GFAP) and pro-apoptotic genes (BAX, IL1ß and KI67) for C6 cells with in groups in various experimental groups. **b** Quantitative analyses of tumor volume** a** and relative tumor weight **c** of the rats in groups in various experimental groups. The *p*-value parameter showed the level of significant differences as. *p < 0.05, **p < 0.01, and # p > 0.05

Apoptosis is mechanism in cell fate and tumor progression through alteration of anti- or pro apoptotic genes which could lead cell death [6, 8, 9, 16, 19, 21, 23]. We have measured several family of proteins includes apoptosis-related molecules including VEGF, BCL2, BAX, IL1-β, BDNF, KI67 genes for treated C6 cells with Ator and MSCs [6, 8, 9, 16, 19, 21, 23]. Meanwhile, both of Ator and MSCs suppressed expression of VEGF, BAX and BDNF rates (Fig. 6a). The BCL2, IL1-β, GFAP and KI67 levels in C6 tumor cells exposed with Ator and MSCs significantly activated compared to control sample (Fig. 6a). The expression of VEGF and PDGF especially at Ator and Ator + MSCs conditions were more than 2.4 times lower than control sample (Fig. 6a). The VEGF and PDGF upregulations are associated to tumor cell development and progression through tumor angiogenesis and growth of blood vessels. Therefore, inhibition of VEGF and PDGF signaling not only blocks angiogenesis in tumors but can also destroy tumor vessels through suppression of cell proliferation and migration [3, 7, 15, 18]. The VEGF and PDGF proteins in treated conditions reflect drug and MSCs activity for tumor inhibition. Furthermore, it is well-know that BCL2 and BAX have to play cardinal roles in regulating mitochondrial translocation and dysfunction as well as oligomerization [3, 7, 15, 18, 21]. The BCL2 gene as an antioxidant and antiapoptotic protein decreased at 1.6–2.4 times for treated conditions with Ator or MSCs and Ator + MSCs conditions compared with non-treated sample (Fig. 6a). The treated conditions with Ator + MSCs possessed lowest rate of IL1β compared with other treated and non-treated conditions. The tumor microenvironment due to modulation of inflammatory cytokines could regulate brain tumor development and progression. The obtained results for IL1β proved tumor supersession which could correlated with results of BCL2 and VEGF proteins [3, 4, 7, 12].

The GBM tumor cells proliferation was associated with KI67 expression. The C6 cells in non-treated cells were expressed in 4 fold KI67 which shows proliferation of C6 cells (Fig. 6a). The utilized Ator, MSCs and combination of these components were able to inhibit C6 cell proliferation as demonstrated by a significant reduction in KI67 labeling index. Interestingly, Ator + MSCs displayed more significant impact on cellular proliferation and KI67 expression index did not changed compared with initial condition. These results for KI67 indexes for experimental conditions could be correlated with cellular growth inhibition measurements [3, 4, 7, 12, 34].

### In vivo analysis

The in vivo study on the C6 induced GBM rat models have cleared that co-administration of Ator with MSCs could significantly increase the animal’s median survival. Moreover, we could detect an abruptly improving the survival of the Ator as well as MSCs treated animals. In following we investigated the antitumor efficacy of Ator and MSCs in implanted GBM tumor model in rats. The experimental implementation scheme was shown in Fig. 1. As can be seen from Fig. 6b, for the PBS treated group (Control), the tumor grew rapidly and its size was highest level compared with other groups. Meanwhile, the Ator and MSCs treated groups showed superior tumor suppressive outcome. Interestingly, the tumor growth of animals treated with simultaneous utilization of Ator and MSCs condition (Ator + MSCs) was dramatically inhibited and the size of tumor was notably smaller than other treated groups. In the same way, as shown in Fig. 6c, the average tumor weight of the treated condition was remarkably smaller than other conditions. The obtained results indicated the satisfactory antitumor effect of Ator and MSCs for GBM model [3, 8, 9, 21, 23]. The simultaneous utilization of Ator and MSCs condition presented superior antitumor efficacy than non-treated condition and single treated animals with Ator or MSCs.

### H&E staining

**Fig. 7:**
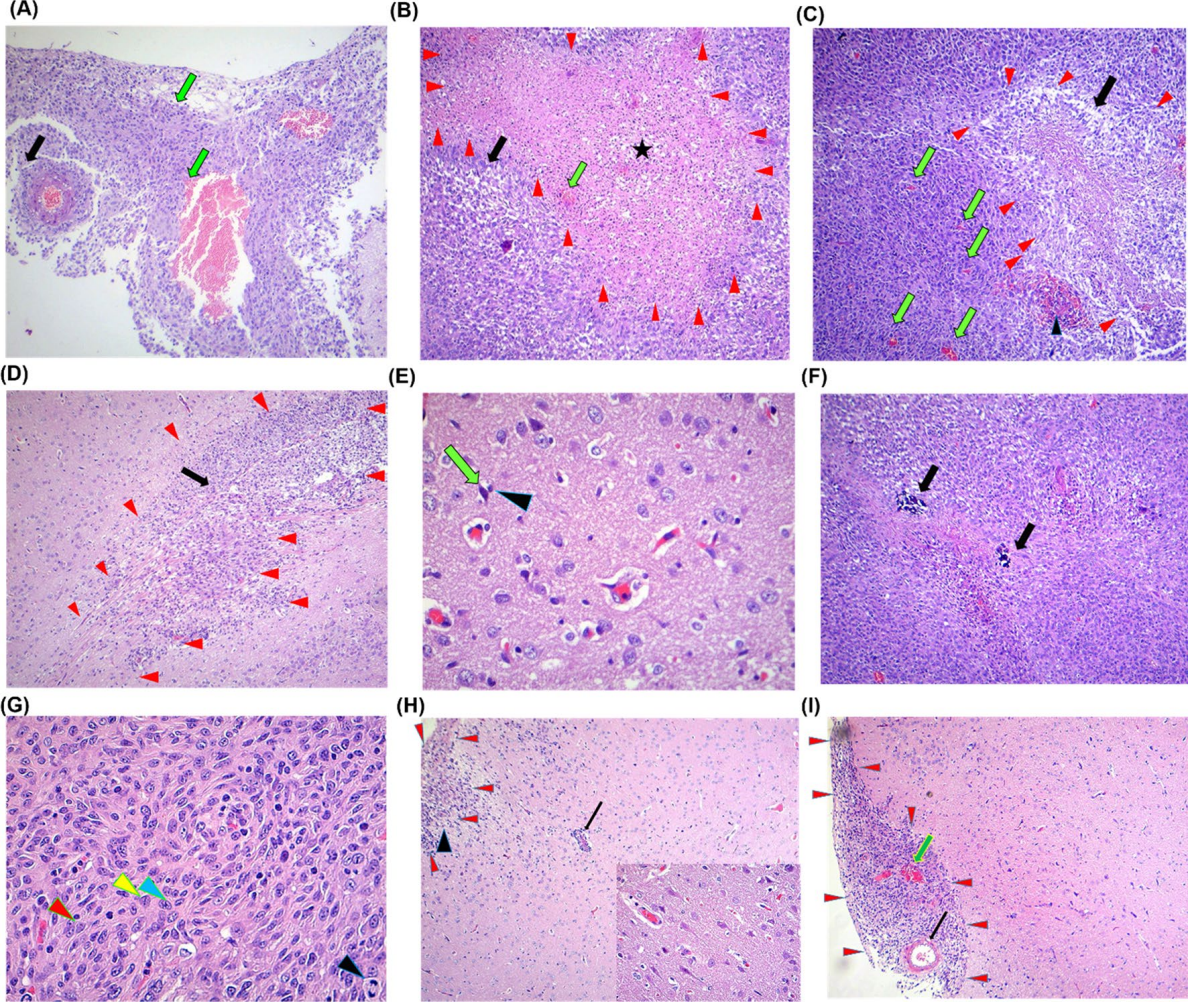
Histopathology of glioblastoma-driven brain tumors. Hematoxylin and eosin staining of brain slice with colored boxes highlighting areas of particular interest for non-treated condition. **a**, **b** and **c** Non-treated condition, **d** and **e** Ator group, **f** and **g** MSCs group and **h** and **i** MSCs + Ator group. **a** Glomeruloid structure is a bizarre form of endothelial proliferation that comprise capillary tufts (black arrow) dispersed amidst a proliferation of endothelial cells and pericytes merged with necrosis, congestion and hemorrhage, **b** Pseudopalisading of neoplastic cells (black arrow) with severe necrosis (star) associated with microvascular proliferation (green arrow), **c** Pseudopalisading with moderate necrosis of neoplastic cells (black arrow) along with microvascular proliferation (green arrows), glomeruloid structures (arrows) dispersed amidst of endothelial cells and pericytes.** d** Pseudopalisading with moderate necrosis of neoplastic cells (black arrow), **e** small delicate vessels characterize the normal vasculature associated with dark neuron (green arrow), microglia cell (arrow head) and moderate cellularity of tumoral cells. **f** This glioblastoma has manifest cellularity, with noticeable hyperchromatism as well as pleomorphism. Note the prominent vascularity and the area of necrosis with neoplastic cells palisading around it, **g** marked cellularity, with marked hyperchromatism, pleomorphic neoplastic cells, multi-nucleoli and (arrow head), high mitotic index and calcification, Inset: four mitosis phases in control group, inset: high mitotic index associated with various mitotic figures including prophase (blue arrow head), metaphase (black arrow head) and anaphase (red arrow head. **h** Low hypercellularity (arrow head), low mitosis index and mild invasive tumor cells (arrow) show a predilection for perivascular cuffing without pseudopalisading necrosis, Inset: normal neuron and small delicate vessels; **i** Glomeruloid (black arrow) and mild or limited invasive tumoral cells associated with microvascular proliferation (green arrows) without pseudopalisading necrosis. (Color figure online)

Following the underlying antitumor mechanism of Ator and MSCs were analyzed by H&E staining and histological analyses of tumor tissues of each conditions. Glomeruloid structure as bizarre form of endothelial proliferation observed for non-treated condition (Control). These structures comprise capillary tufts (black arrow) which dispersed amidst a proliferation of endothelial cells and pericytes associated with necrosis as well as congestion and hemorrhage region (Fig. 7a).

The several phenomena including pseudopalisading with moderate necrosis of neoplastic cells, small vessels distinguish the normal vasculature associated with dark neuron, existence of higher number of microglia cells and moderate cellularity of tumoral cells validate anti-angiogenesis and apoptotic activity of Ator on GBM in the rat model (Control) (Fig. 7b). Moreover, the moderate pseudopalisading necrosis of neoplastic cells along with growth of microvascular and glomeruloid structures distributed amidst a proliferation of endothelial cells and pericytes which observed in non-treated condition (Control) (Fig. 7c). As shown in Fig. 7d and e in Ator group the moderate pseudopalisading necrosis of neoplastic cells, and formation of small delicate vessels representing vascularity in normal form which associated with dark microglia cell and moderate cellularity of tumoral cells in this group. For MSCs goup, this glioblastoma has presented high cellularity, with mild hyperchromatism as well as pleomorphism of cells and nuclei. Besides, the vascularity as well as necrosis region with neoplastic cells palisading around it observed for MSCs treated group (Fig. 7f). In addition, existence of the cellularity, hyperchromatism, pleomorphic neoplastic cells, multi-nucleoli and high mitotic index and calcification displayed for MSCs group. Interstingly, in Ator + MSCs group the low hypercellularity and mitosis index as well as mild level of invasive tumor cells show a predilection for perivascular cuffing and inflammation around a blood vessel without pseudopalisading necrosis (Fig. 7h). Morever, glomeruloid structure and mild or limited invasive tumoral cell density accompanied with proliferation of microvascular without pseudopalisading necrosis were indicative in Ator + MSCs group (Fig. 7i).

The anti-angiogenesis and apoptotic activity of Ator against GBM cell line has been previously reported by Bayat et al. [3]. In MSCs group, low hypercellularity and mitosis index as well as mild invasive of tumor cells without pseudopalisading necrosis indicate that the anticancer effect of MSCs in a rat model of GBM. It has been shown that by means of tumour-specific tropism of MSCs can be transduced to deliver anticancer agents such as interleukins (IL-2, IL-7, IL-18, and IL-12), TRAIL, and interferon (IFN-β and IFN-γ) directly to glioma sites to kill tumour cells or to regulate immune responses [2, 4, 35, 36]. Mild or limited invasive tumoral cells associated with low microvascular proliferation without pseudopalisading necrosis, in Ator + MSCs group shows that simultaneous utilization of MSCs and Ator can exert more anticancer activity against GBM rather than MSCs and Ator alone in experimental GBM model.

## Conclusions

Whether adding Ator and MSCs could improve anti-tumorigenicity in glioblastma tumor and inhibit tumor cell proliferation was evaluated in vitro and in vivo studies. It could be demonstrated that the co-treatment of Ator with MSCs could augment the cytotoxic effects of Ator on the C6 cell lines. Their multipotency, pro-regenerative effects, immunoregulatory and their chemotactic properties to home to tumor invaded regions make them a good candidate for cancer therapy.

## Data Availability

The datasets used and/or analyzed during the current study are available from the corresponding author on reasonable request.

## Abbreviations

GBM: Glioblastoma multiform
CNS: Central nervous system
MSCs: Mesenchymal stem cells
Ator: Atorvastatin
IL-1β: Interleukin-1β
IL-6: Iinterleukin-6
IL-8: Interleukin-8
MAPK: Mitogen-activated protein kinase
VEGF: Vascular endothelial growth factor
TNF-α: Tumor necrosis factor-α
TGF-β1: Transforming growth factor-β1
H&E: Hematoxylin and eosin
